# Robotic Excision of an Incidental Urachal Mucinous Cystadenocarcinoma in a Patient With Concomitant Endometrial Carcinoma

**DOI:** 10.7759/cureus.78741

**Published:** 2025-02-08

**Authors:** William A Langbo, Jamie Yoon, Han Hee D Kim, Katherine M Sinchek, Alexander K Chow

**Affiliations:** 1 Department of Urology, Rush University Medical Center, Chicago, USA

**Keywords:** adenocarcinoma, bladder cancer, endometrial carcinoma, robotics, urachal cancer, urachus

## Abstract

Urachal cystadenocarcinoma (UC) is a rare but highly aggressive subtype of bladder cancer. While most localized cases of UC are asymptomatic, occasionally, patients present with nonspecific lower urinary tract symptoms. Unfortunately, most cases are not detected until advanced disease is present. The presence of local nodal or distant metastasis is particularly important for prognosis, drastically reducing five-year overall survival rates. The gold standard for localized UC is wide surgical excision, with no established role of chemotherapy. On the contrary, endometrial carcinoma (EC) is the most common gynecologic malignancy. As such, staging, prognosis, and treatment guidelines of endometrial carcinoma are well-established. The gold standard treatment for localized endometrial carcinoma includes total abdominal hysterectomy and bilateral salpingo-oophorectomy (TAH-BSO) and lymph node dissection (LND). Concurrent cases of urachal cystadenocarcinoma and endometrial carcinoma are exceedingly rare. To the authors’ knowledge, there have been no known reports of these co-occurring malignancies thus far in the literature. We present the case of a 66-year-old patient with nonspecific lower urinary symptoms and incidentally discovered UC in the setting of concurrent endometrial carcinoma. The patient underwent robotic excision of the urachal cystadenocarcinoma and TAH-BSO, followed by adjuvant chemotherapy and vaginal brachytherapy. Surveillance imaging at 16 weeks after surgery showed no evidence of disease recurrence.

## Introduction

Urachal cystadenocarcinoma (UC) is a rare and aggressive form of bladder cancer, accounting for approximately 0.01% of adult cancers and 1% of bladder malignancies [1]. Urachal cystadenocarcinoma typically has a nonspecific presentation, delaying detection until progression to advanced disease [1]. However, UC occasionally presents with lower urinary tract symptoms [2]. It is often found incidentally on computed tomography (CT) or magnetic resonance imaging (MRI), and diagnosis can be confirmed with a biopsy. Wide surgical excision is the gold standard treatment for localized UC. Early recognition and prompt treatment are key as the mean survival for locally advanced or metastatic disease ranges from 12 to 24 months, with a five-year survival rate of less than 20% [1].

Endometrial carcinoma (EC) is the fourth most common malignancy in women in the United States [3]. With approximately 142,000 new cases annually and an incidence that is expected to rise, EC often presents with abnormal uterine bleeding in post-menopausal women [4]. Approximately 75% of cases of EC are diagnosed at an early stage, and the prognosis is excellent, with an overall combined five-year survival of 80% [3,4]. The treatment of localized disease most often consists of surgical resection, including total abdominal hysterectomy and bilateral salpingo-oophorectomy (TAH-BSO) and pelvic lymph node dissection (LND) [4]. Adjuvant chemotherapy, most commonly consisting of a regimen of carboplatin and paclitaxel, is reserved for diseases with positive lymph nodes, as well as certain high-risk subtypes (including serous carcinoma) of localized disease [5]. Despite the frequency with which endometrial carcinoma is diagnosed, concurrent endometrial carcinoma and urachal cystadenocarcinoma are exceptionally rare, with no known reports in the literature to date. We report a case of concurrent primary urachal cystadenocarcinoma and primary endometrial carcinoma in a patient who underwent consecutive robotic urachal mass excision and TAH-BSO.

## Case presentation

**Figure 1:**
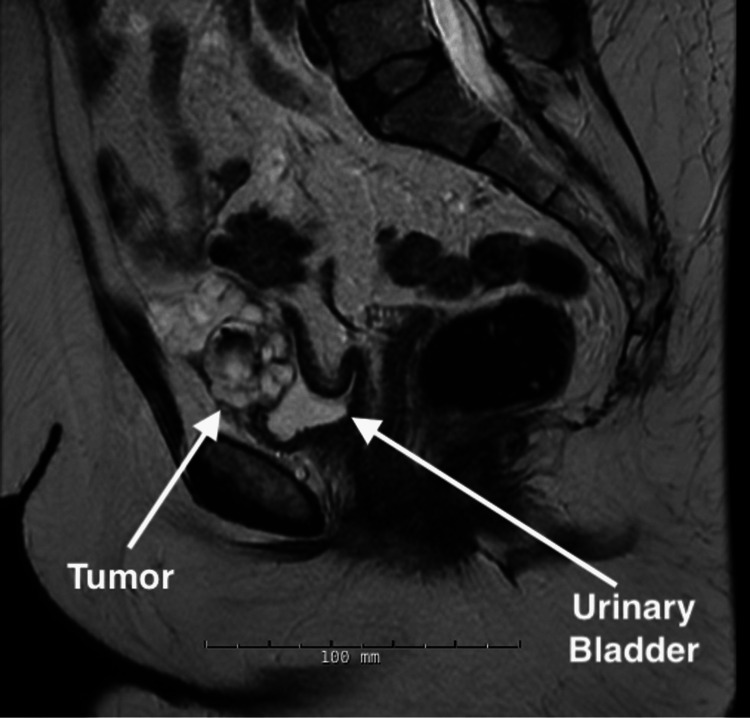
Sagittal MRI depicting the urachal mucinous cystadenocarcinoma in relation to the urinary bladder. MRI: magnetic resonance imaging

**Figure 2:**
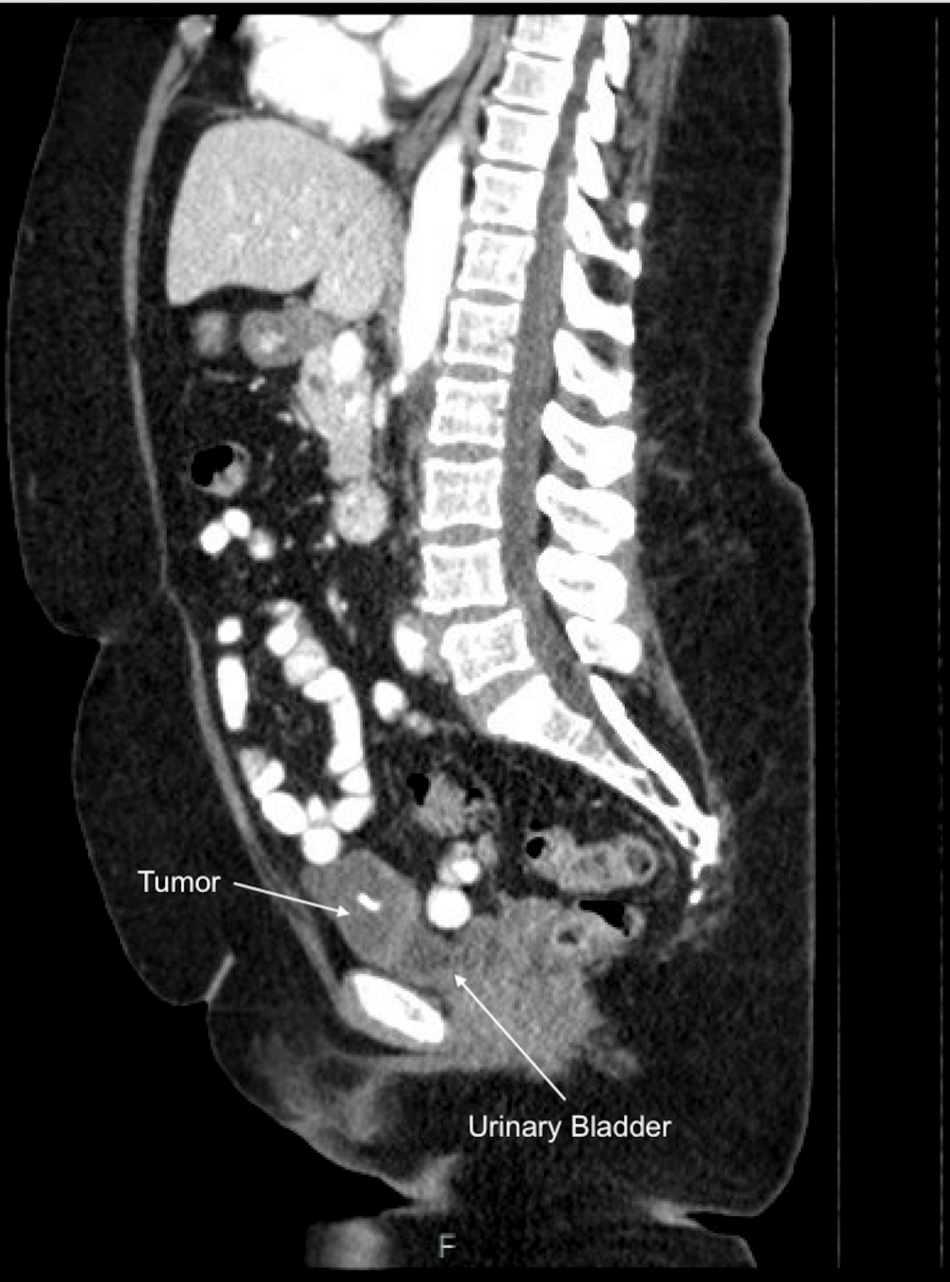
Sagittal CT of the abdomen and pelvis re-demonstrating the urachal mucinous cystadenocarcinoma in relation to the urinary bladder. CT: computed tomography

A 66-year-old woman with an 11.25-pack-year tobacco history and no family history of cancer or prior abdominal or pelvic surgeries presented with five months of post-menopausal abnormal uterine bleeding and chronic urinary urgency. A pap smear was obtained and revealed glandular cell abnormalities, positive for adenocarcinoma. Subsequent endometrial biopsy was positive for serous endometrial intraepithelial carcinoma with a consistent immunoprofile. A diagnostic MRI incidentally revealed a suspicious mass on the anterior dome of the bladder (Figure 1). Cystoscopy showed a tumor on the anterior dome of the bladder with central erosion into the bladder lumen. A subsequent CT of the abdomen and pelvis re-demonstrated a multi-septated, lobulated, exophytic lesion arising from the anterior urinary bladder dome (Figure 2). The patient elected to proceed with robot-assisted wide resection of the urachal mass and partial cystectomy with concomitant total abdominal hysterectomy and bilateral salpingo-oophorectomy (TAH-BSO).

A multidisciplinary surgical team was assembled consisting of urologic and gynecologic oncology surgeons. The patient was positioned in dorsal lithotomy and prepped and draped in the usual sterile fashion. Eight-millimeter trocars were placed in the midline superior to the umbilicus, at the left and right rectus margin slightly caudal to the umbilicus, medial to the right anterior-superior iliac spine, and an 8 mm AirSeal (ConMed, Utica, NY) assistant port was placed medial to the left anterior-superior iliac spine. The patient was placed in a steep Trendelenburg position, and the da Vinci Xi robot (Intuitive Surgical, Sunnyvale, CA) was docked. The peritoneum was inspected, and there were no abnormalities or adhesions noted. The urachus was identified and incised at the level of the umbilicus. The peritoneum was incised lateral to the medial umbilical ligaments, and the incisions were carried laterally toward the round ligaments to mobilize the urachus and bladder. The urachus was dissected to the level of the bladder, and a full-thickness cuff of the bladder was excised en bloc with the urachus, including the previously described lesion at the bladder dome, with the aid of cystoscopic visualization. The gynecologic oncology team then proceeded with standard TAH-BSO with pelvic lymph node dissection. All specimens were extracted through the vaginal cuff.

**Figure 3:**
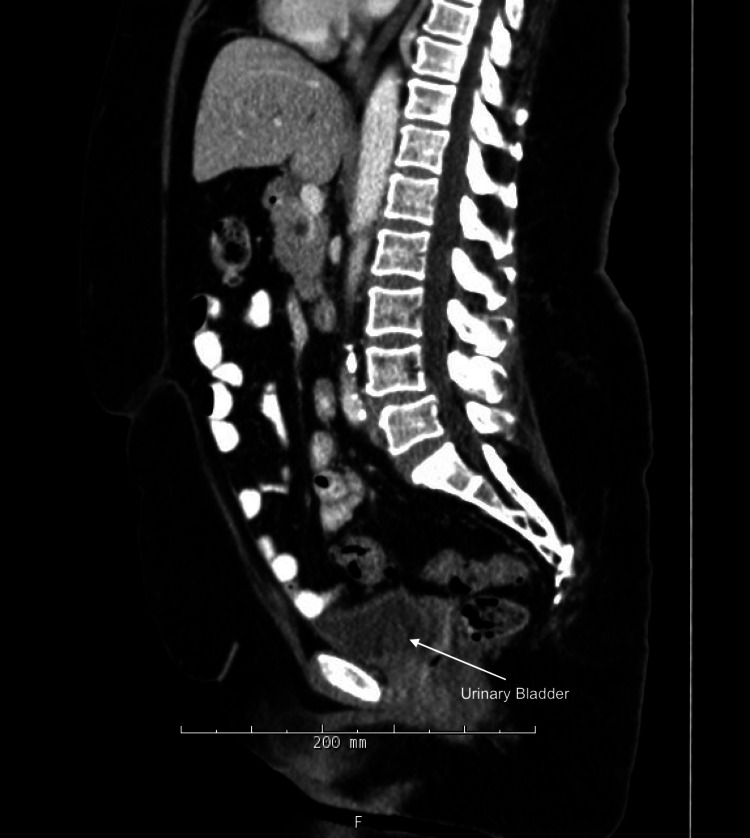
Postoperative sagittal CT of the abdomen and pelvis demonstrating no evidence of disease recurrence. CT: computed tomography

The histopathologic evaluation of the mass confirmed primary urachal mucinous cystadenocarcinoma measuring 7.9 × 5.7 × 3.5 cm, stage pT2Nx, staining positive for cytokeratin (CK) 7, CK20, and caudal-related homeobox transcription factor-2 (CDX-2). The tumor was reported to be less than 0.1 cm from the serosal surface of the specimen and involved the muscularis wall of the urachus. Pathology was not able to determine the orientation of the specimen and thus could not comment on tumor margins. Per National Comprehensive Cancer Network guidelines, no adjuvant therapy was recommended. The evaluation of the uterus and cervix confirmed serous endometrial intraepithelial carcinoma, measuring 1.9 cm, stage pT2N0. There was no myometrial or uterine serosal involvement; however, the tumor did involve the cervical stroma; 0/2 pelvic lymph nodes were positive for the disease. The patient underwent six cycles of adjuvant carboplatin/paclitaxel chemotherapy in addition to high-dose-rate vaginal brachytherapy. CT scan at six months from surgery, following adjuvant treatment, demonstrated no evidence of disease recurrence (Figure 3).

## Discussion

Urachal cystadenocarcinoma is a rare diagnosis associated with high mortality. Furthermore, the presence of concurrent UC and endometrial carcinoma is exceedingly rare, with no reports available thus far in the literature. The existing literature emphasizes the importance of early detection, diagnosis, and treatment to optimize survival. The rarity of UC places the diagnosis at risk of being overlooked; however, UC is a “can’t-miss” diagnosis in a patient with an anterior bladder dome lesion. Furthermore, in the presence of an additional pelvic malignancy, UC further risks being overlooked or misidentified as a possible metastasis. Other differential diagnoses of an anterior bladder dome lesion to consider include villous adenoma and mucinous cystic tumor of low malignant potential. In patients with lesions concerning for urachal cystadenocarcinoma, biopsy with pathologic evaluation is crucial for diagnosis. Distinctive findings in urachal cystadenocarcinoma include the presence of papillary or cribriform structures with focal or diffuse severe nuclear atypia and high levels of mitoses [6]. To differentiate primary from secondary tumors, immunohistochemical stains CK7, CK20, and CDX-2 along with 34βE12 and β-catenin can be utilized [6]. While on rare occasions urachal cystadenocarcinomas can metastasize to the reproductive organs, metastases are grossly and microscopically consistent with the primary tumor. The most common site of distant metastasis is the peritoneum [1]. Thus, the laparoscopic evaluation of the peritoneum is important for the proper assessment of the extent of the disease.

The presence of metastasis is the most important prognostic factor, with five-year survival dropping from approximately 80% to less than 20% in patients with local or distant metastasis [1]. Additional factors including tumor stage and larger tumor size at the time of identification indicate a worse prognosis in patients with UC. Furthermore, previous studies have shown no significant difference in five-year overall survival between local lymph nodes and distant metastatic disease [1]. The role of lymphadenectomy remains debated, with limited data showing no significant advantage in overall survival in patients who underwent lymphadenectomy [1]. The option of sentinel lymph node biopsy has been described for UC; however, it lacks significant evidence relative to standard lymphadenectomy data [7]. In the presence of concurrent endometrial carcinoma, lymphadenectomy is performed at the time of TAH-BSO for the diagnostic evaluation and staging of EC. Adjuvant chemotherapy with a carboplatin/paclitaxel regimen is recommended for patients with stage IIIC (involving the lymph nodes) or high-risk stage I endometrial carcinoma. It is unknown whether these chemotherapies might have any effect on the recurrence of localized UC; however, a similar platinum-based chemotherapy, cisplatin, is being investigated in metastatic UC. There is no currently established chemotherapy regimen for metastatic UC, although recent studies have shown promising results with 5-fluorouracil/cisplatin regimens, as well as emerging immunotherapies [2,8]. In summary, early detection, identification, and wide surgical excision are essential in conferring favorable survival outcomes in patients with UC. In the presence of concurrent EC, TAH-BSO with diagnostic lymphadenectomy can be performed at the time of UC excision to promptly treat and stage both primary malignancies.

## Conclusions

While rare, urachal cystadenocarcinoma is a highly aggressive malignancy, and care should be taken not to overlook this diagnosis among other probable etiologies. In the extremely rare setting of concurrent high-risk endometrial carcinoma, it is important to properly identify both primary malignancies to guide further treatment. Wide surgical resection remains the gold standard for localized urachal cystadenocarcinoma. TAH-BSO with adjuvant chemotherapy and vaginal brachytherapy is the gold standard for localized high-risk endometrial carcinoma with cervical involvement. The accurate diagnosis of both primary malignancies remains key in ensuring proper treatment and favorable outcomes. Early intervention is imperative, as locally advanced and metastatic UC has extremely low survival rates.
